# Gender Differences in Genetic Associations of RAB38 with Urinary Protein-to-Creatinine Ratio (UPCR) Levels in Diabetic Nephropathy Patients

**DOI:** 10.3390/jpm10040184

**Published:** 2020-10-21

**Authors:** Zhi-Lei Yu, Chung-Shun Wong, Yi Ting Lai, Wan-Hsuan Chou, Imaniar Noor Faridah, Chih-Chin Kao, Yuh-Feng Lin, Wei-Chiao Chang

**Affiliations:** 1 Department of Clinical Pharmacy, School of Pharmacy, Taipei Medical University, Taipei 110301, Taiwan; r06a41003@ntu.edu.tw (Z.-L.Y.); s8901752004@gmail.com (Y.T.L.); ocean.chou@tmu.edu.tw (W.-H.C.); imaniar.faridah@pharm.uad.ac.id (I.N.F.); 2 Graduate Institute of Clinical Medicine, College of Medicine, Taipei Medical University, Taipei 110301, Taiwan; johnson7617@gmail.com; 3 Department of Emergency Medicine, Taipei Medical University-Shuang Ho Hospital, New Taipei City 235041, Taiwan; 4 Department of Emergency Medicine, School of Medicine, College of Medicine, Taipei Medical University, Taipei 11031, Taiwan; 5 Faculty of Pharmacy, Ahmad Dahlan University, Yogyakarta 55164, Indonesia; 6 Division of Nephrology, Department of Internal Medicine, Taipei Medical University Hospital, Taipei 110301, Taiwan; salmonkao@gmail.com; 7 Division of Nephrology, Department of Internal Medicine, School of Medicine, College of Medicine, Taipei Medical University, Taipei 110301, Taiwan; 8 Division of Nephrology, Department of Internal Medicine, Shuang Ho Hospital, Taipei Medical University, New Taipei City 235041, Taiwan; 9 Master Program for Clinical Pharmacogenomics and Pharmacoproteomics, School of Pharmacy, Taipei Medical University, Taipei 110301, Taiwan; 10 Integrative Research Center for Critical Care, Wan Fang Hospital, Taipei Medical University, Taipei 110301, Taiwan; 11 Department of Medical Research, Shuang Ho Hospital, Taipei Medical University, New Taipei City 235041, Taiwan

**Keywords:** DM (diabetes mellitus), DN (diabetic nephropathy), RAB38, albuminuria, genetic polymorphism

## Abstract

Renal dysfunction is common in patients with diabetes mellitus (DM). Previous findings from a meta-analysis of GWAS indicated that the variation of *RAB38/CTSC* is highly associated with the urinary albumin-to-creatinine ratio (UACR) in European populations. In addition, *RAB38* knockout rats showed an increase in urinary albumins. Although the prevalence of chronic kidney disease is high in Taiwan, the role of genetic variants in diabetic renal function is still unclear. In the current study, 275 diabetic nephropathy (DN) patients were recruited to perform a genetic association study. Our results indicated that rs1027027, rs302647, and rs302646 in *RAB38* were significantly associated with urinary protein-to-creatinine ratio (UPCR) levels in DN patients. Importantly, after analysis stratified by gender, a significant genetic influence on UPCR levels was observed in the male population. The findings confirmed the roles of gender and variants of *RAB38* in the risk of UPCR in Diabetic Nephropathy patients.

## 1. Introduction

Diabetes mellitus (DM) affects around 9.3% of all adults worldwide [1]. Renal dysfunction is common in patients with DM, which is the leading cause of chronic kidney disease (CKD) and end stage renal disease (ESRD) in Taiwan [2]. Diabetic nephropathy (DN) is characterized by glomerular hypertrophy, proteinuria, and renal fibrosis that resulted in the loss of renal function. The appearance of albuminuria is a hallmark of DN. Elevation of albuminuria is associated with an increased risk for CKD progression and ESRD [3]. Several mechanisms of DN have been proposed such as: (1) abnormal lipid metabolism, (2) glomerular hyperfiltration, (3) defected podocyte-specific insulin signaling, (4) congenital mutation in nephrin expression, (5) advanced glycation end products (AGEs), (6) hyperglycemia, (7) activation of cytokines, and (8) vascular endothelial growth factors (VEGFs) signaling [3,4,5,6,7,8,9,10]. Numerous familial aggregation studies have indicated that genetic factors are involved in the development and progression of DN [11,12]. Indeed, genetic factors have been considered to be relevant to the etiology of DN [8]. Epidemiologic studies also indicated that neither the presence of hyperglycemia nor genetic variants alone are sufficient to elicit the renal damage that typically manifests itself as albuminuria in diabetes [13,14].

Genome-wide association studies (GWASs) have been widely applied to identify susceptibility genes that associated with the risk of diabetic kidney diseases [15,16,17,18,19,20]. For example, Alexander et al. performed a meta-analysis of GWAS that nicely demonstrated the strong correlation between the variation of *RAB38/CTSC* and the urinary albumin-to-creatinine ratio (UACR). Furthermore, streptozotocin-induced diabetic *RAB38* knockout rats expressed higher urinary albumin concentrations and decreased amounts of megalin and cubilin at the proximal tubule cell surfaces [20]. The results suggested a critical role of the *RAB38* signaling pathway in the pathogenesis of DN. Because of the high incidence and prevalence of the ESRD in Taiwan, the aim of this study is to determine the genetic role of *RAB38* for diabetic nephropathyin a Taiwanese population.

## 2. Materials and Methods

### 2.1. Study Subjects

**Figure 1:**
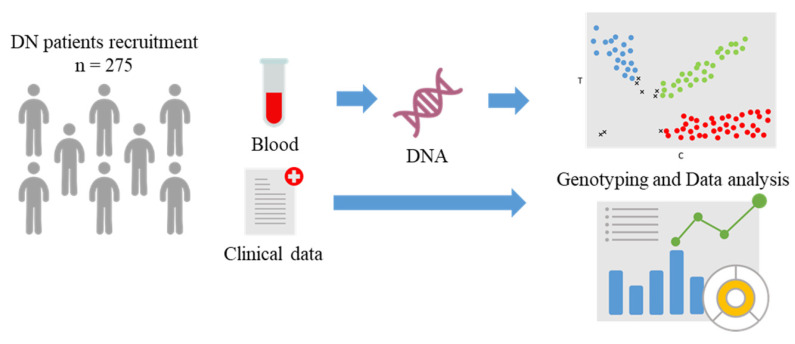
A workflow for Genetic Association Studies.

A schematic depiction illustrated the study workflow in Figure 1. A total of 275 patients were recruited from Taipei Medical University-Shuang Ho Hospital between January 2015 to December 2016 and Taipei Medical University Hospital between January 2014 to May 2015. Inclusion criteria were age between 20 and 90 years, patients with a confirmed diagnosis of DM (fasting glucose above 126 mg/dL or non-fasting glucose above 200mg/dL) before the onset of CKD (presence of albuminuria or eGFR below 60 mL/min/1.73 m^2^). Overt proteinuria was defined as the level that is easily detectable using routine screening methods (usually greater than 300 to 500 mg/day). Overt albuminuria, macroalbuminuria, or proteinuria is defined as a total daily protein excretion >300 mg and <3.5 g. Nephrotic range proteinuria is defined as a total daily protein excretion of >3.5 g (random urine protein/urine creatinine ratio >3.5). Chronic kidney disease is defined as having two previous estimated Cockcroft and Gault creatinine clearance values (eGFR) <60 mL/min per 1.73 m^2^ or eGFR >60 mL/min per 1.73 m^2^ with kidney damage 3 to 6 months apart. Estimated GFR (mL/min/1.73 m^2^) = 186.3 * (Creatinine/88.4)^−1.154^ * (Age)^−0.203^ * (0.742 if female) * (1.210 if black) [21]. Patients with obvious infectious disease, as well as those who underwent kidney transplantation or surgery within 3 months preceding the study were excluded. The detailed clinical data including age, sex, body mass index, smoking, comorbidities status and laboratory parameters were collected. The formulation of urinary protein–creatinine ratio (UPCR) is as follows: UPCR (mg/g) = (urine total protein, mg/dL) * 1000/(urine creatinine, mg/dL). This study conformed to the Declaration of Helsinki, and the study protocol was approved by the regional ethics committee at Taipei Medical University (TMU-JIRB No.: 201411056). Written informed consent from all subjects were received before any data were collected.

### 2.2. Selection of Candidate Single-Nucleotide Polymorphisms (SNPs)

A previous study indicated that *RAB38* is a critical gene for DN-mediated kidney function. This finding was validated by RAB38 knockout mice experiments [20]. Tagging SNP (tSNP) is a representative SNP in a region of the genome with high linkage disequilibrium. In this study, the Genomes Browser Ensembl GRCh37 release 87 was used to download the information of Han Chinese in Beijing (CHB) from 1000 Genome Project (Dec 2016 (phase 3)). A total of 10 tSNPs were selected through the Haloview 4.2 program at r^2^ greater than or equal to 0.7. The Hardy–Weinberg p cutoff was set at 0.01. The minor allele frequency (MAF) of these tSNPs in the Taiwanese population was verified to be greater than 10% on the website of Taiwan Biobank: https://taiwanview.twbiobank.org.tw/index (accessed on 30 April 2020). However, the primer of rs3740925 failed to pass the quality control and there was no substitute SNP, thus nine SNPs were used to conduct genotyping in this study. A pairwise LD map for RAB38 was constructed with a CHB panel using the R package *LDheatmap* (Figure S1).

### 2.3. DNA Extraction

Whole blood was collected from patients who met the inclusion criteria. Genomic DNA was isolated from the buffy coat using the standardized method in the Gentra Puregene kit (QIAGEN, Hilden, Germany). A typical extraction yields approximately 100 ug of DNA from 3 mL of whole blood.

### 2.4. Genotyping

Nine SNPs were genotyped using TaqMan SNP Genotyping Assays (Applied Biosystems, Foster City, CA, USA). Briefly, Taqman probes are labeled with different fluorescent markers. PCR primers and TaqMan probes are designed with SNP sites. Reaction was performed in 96 well microplates with ABI 9700 thermal cycles (Applied Biosystems, Foster City, CA, USA). Fluorescence was measured by the ABI Real Time PCR system and analyzed with StepOne software version 2.22 (Applied Biosystems, Foster City, CA, USA).

### 2.5. Statistical Analysis

All statistical analysis was conducted by using R software (version 3.6.0, Vienna, Austria). Spearman’s correlations between clinical variables were calculated with an R *corr* package. Associations between UPCR and genotypes were analyzed under genotypic, dominant, recessive, and log-additive models with the R *SNPassoc* package. Furthermore, associations between DN susceptibility and genotypes were analyzed using Fisher’s exact test. Any difference with *p* value < 0.05 was considered as statistically significant. The q values as the false discovery rate (FDR) estimation with the Benjamini–Liu method were calculated using the R *discreteMTP* package for multiple-testing correction [22]. The Genotype-Tissue Expression (GTEx) V8 database was interrogated to investigate the correlations between genotypes of the UPCR-associated variants and tissue-specific *RAB38* expression (accession date: 22 June 2020). Moreover, the statistical power for the analysis of association between SNPs and DN susceptibility was calculated using the GASpower calculator. It is an interactive tool to calculate power for genetic association studies in case-control designs [23].

### 2.6. General Population in Taiwan Biobank

The Taiwan Biobank is a biological database exclusively for the Taiwanese general population. This is a large-scale community-based cohort of relatively healthy volunteers who either have no history of cancer or are self-claimed cancer-free, and a hospital-based cohort including patients affected by pervasive chronic diseases as well as cancer patients. Currently, the Taiwan Biobank has accumulated over 114,000 voluntary participants. Among them, DNA of 20,645 participants was subjected to genome-wide genotyping using Affymetrix Axiom TWB array, and 1000 participants were sequenced with the Illumina platform. The summary statistics of their variants is available online. In this study, tSNPs allele and genotype frequencies from the genome-wide genotyping and sequencing data in the community-based cohort were queried in the Taiwan Biobank (https://www.twbiobank.org.tw/new_web/index.php (accessed on 30 April 2020)).

## 3. Results

### 3.1. Demographic and Clinical Characteristics of Study Samples

**Figure 2:**
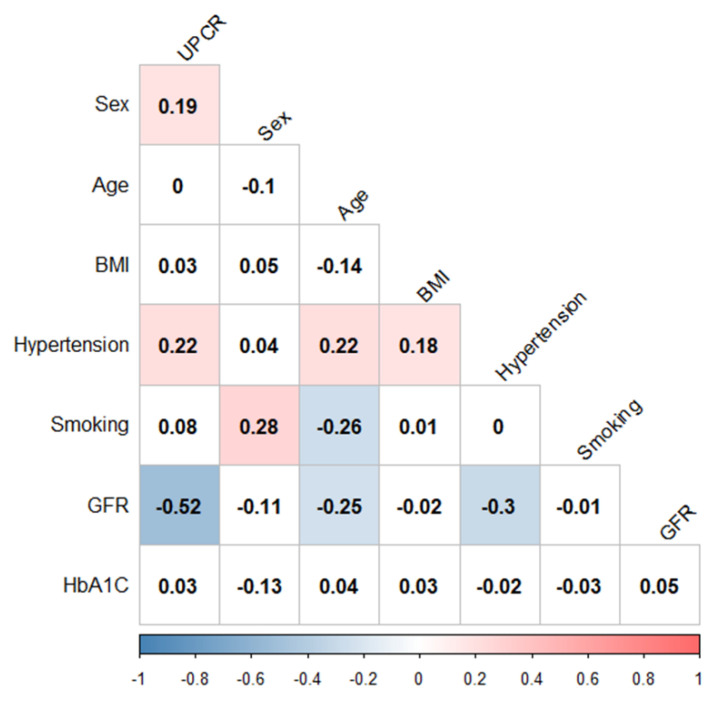
Spearman correlation plot of clinical characteristics in diabetic nephropathy (DN) patients. Significant positive and negative correlations (*p* < 0.01) are shown in red and blue, respectively. Spearman’s correlation coefficients are shown in wells. UPCR: urine protein and creatinine ratio; BMI: body mass index; GFR: glomerular filtration rate; HbA1C: hemoglobin A1c.

A total of 275 patients were recruited in this study. Among these patients, 57.45% are male and 42.55% are female. The mean age was 66 years old. GFR was 53.17 ± 34.71 mL/min/1.73 m^2^. The mean UPCR was 1723.67 mg/g (Table 1). In Figure 2, a Spearman correlation plot demonstrates the relevance between various clinical characteristics in this diabetic nephropathy study cohort. We found that UPCR has a significant positive correlation with sex (Spearman correlation coefficient: 0.19) and hypertension (Spearman correlation coefficient: 0.22), and it is negatively correlated with eGFR (Spearman correlation coefficient: −0.52).

### 3.2. rs1027027, rs302647, rs302646 Are Associated with UPCR Levels in DN Patients

**Figure 3:**
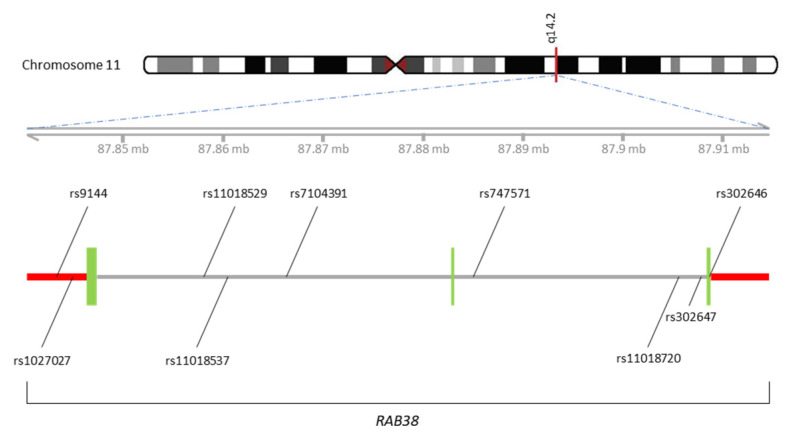
Illustration of genotyped human *RAB38* gene polymorphisms on chromosome 11. Red bar represents untranslated regions (UTR). Green bar represents exons.

For this study, nine tagging SNPs of *RAB38* were selected. The relative locations of the SNPs on chromosome 11 were depicted in Figure 3. Of these tSNPs, rs9144 and rs1027027 are located on 3′untranslated region (3′-UTR), rs302646 is located on the exon, and the others are in intron regions. The position, location, alteration and allele frequencies of nine tSNPs among different populations are illustrated in Table 2. Allele frequencies of the Taiwanese population were queried from the array and sequencing data in the community-based cohort of the Taiwan Biobank. Similar allele frequency in Taiwan biobank and current cohort indicated a good genotyping quality in this study. The frequencies of all SNPs were in Hardy–Weinberg equilibrium.

We analyzed the association between SNP and UPCR. As shown in Table 3, after analysis adjusted for possible covariates such as age, gender, GFR and hypertension, rs1027027, rs302647, and rs302646 revealed a significant association with UPCR level under the log-additive model after FDR control for tests on multiple SNPs (q = 0.027, 0.033, 0.018, respectively). In rs1027027, C/C genotype showed the lowest mean UPCR of 1487.4 ± 165.3 mg/g, and with the increment of A allele, UPCR elevated correspondingly. The patients with C/A and A/A genotypes at rs1027027 have UPCR levels of 2605.4 ± 549.1 and 4475.5 ± 3295.8 mg/g, respectively. For rs302647, the patients with C/C genotype showed the lowest mean UPCR of 1477.8 ± 207.6 mg/g, and patients with the increment of G allele, UPCR elevated. The C/G genotype had a mean UPCR of 1950.1 ± 314.9 mg/g and the GG genotype had the highest mean UPCR of 2365.4 ± 697.4 mg/g. For rs302646, the A/A genotype showed the lowest mean UPCR of 1126.9 ± 268.3 mg/g, and UPCR elevated as the G allele incremented. The A/G genotype had a mean UPCR of 1404.8 ± 197.9 mg/g and the G/G genotype had the highest mean UPCR of 2440.0 ± 376.9 mg/g. Moreover, the distribution of lnUPCR in different genotypes at rs1027027, rs302647, and rs302646 showed no significant differences between groups (Figure S2). 

### 3.3. Gender Difference in the Association between rs1027027, rs302647, rs302646 and UPCR Levels in DN Patients

Furthermore, we conducted a subset analysis stratified by gender. Strikingly, a significant difference in the genetic association of UPCR was observed (Table 4 and Table 5). Among the nine SNPs examined, rs1027027, rs302647, and rs302646 in the male cohort were significant under the log-additive model (q = 0.032, 0.045, and 0.032). However, no significant differences were found in the female cohort.

### 3.4. rs1027027, rs302647, rs302646 Are Not Associated with DN Susceptibility

To investigate the correlation between DN susceptibility and the UPCR-associated polymorphisms, we compared the genotype frequencies and allele frequencies between this study cohort and the general population from the Taiwan Biobank. For rs1027027, the genotype and allele frequencies were queried from the GWAS data (*n* = 24,625). For rs302647 and rs302646, for which the genome-wide genotyping data were not available, the genotype and allele frequencies data were queried from the DNA sequencing data (*n* = 1000). The association between SNPs and DN susceptibility was tested using Fisher’s exact test. However, no apparent differences were found. The results indicated that variants in RAB38 are not associated with DN susceptibility (Tables S1–S3). 

Statistical power for DN susceptibility analysis was calculated by a GASpower calculator using the following parameters: Numbers of cases/control were 265/24625 for rs1027027, 255/1000 for rs302647, and 267/1000 for rs302646. The significance level of this study design was 0.0167. Prevalence of DN was 0.179 based on a previous study [24]. Disease allele frequencies were 0.119, 0.282, and 0.559 for rs1027027, rs302647, and rs302646, respectively. Furthermore, the genotype relative risk (GRR) is calculated based on the disease model for each SNP. The GRR in the dominant model were 0.811, 1.253, and 1.066 for rs1027027, rs302647, and rs302646, respectively. The GRR in the recessive model were 0.922, 1.324, and 1.130 for rs1027027, rs302647, and rs302646, respectively. Results further showed that the statistical power is inadequate for the susceptibility of DN. The statistical power of rs1027027, rs302647, and rs302646 was 0.017, 0.256, and 0.024 in the dominant model, and 0.017, 0.078, and 0.066 in the recessive model, respectively.

### 3.5. rs1027027, rs302647, rs302646 Are Associated with RAB38 Expression in Different Tissues

To explore the functional role of UPCR-associated variants, we interrogated GTEx database V8 for the eQTL effects of these variants on *RAB38* expression in different tissues. As shown in Table S4, the A allele at rs1027027, which was associated with higher UPCR levels in DN patients, is correlated with decreased *RAB38* expression in esophagus mucosa. Similarly, the G allele at rs302646 is associated with lower RAB38 expression in various tissues. However, for rs302647, the G allele has a positive effect on *RAB38* expression in a variety of tissues, including adipose, colon, nerve, heart, lung, and whole blood.

## 4. Discussion

Previous meta-analysis of GWAS demonstrated that variation of RAB38/CTSC was highly associated with UACR of European ancestry with diabetes [20]. Urine albumin–creatinine ratio (UACR) and urine protein–creatinine ratio (UPCR) are important markers of kidney damage. Both UACR and UPCR have been utilized for prognosis in persons with chronic kidney disease (CKD). Several studies demonstrated that UACR and UPCR are relatively comparable in their associations with common complications of CKD. Furthermore, they have similar predictive values of clinical endpoints, including all-cause mortality, initiation of renal replacement therapy, doubling of serum creatinine, and > 30% decline in eGFR [25,26,27]. However, UPCR showed a superior performance to UACR in the prediction of 24-h proteinuria and that can identify patients with high non-albumin proteinuria, who otherwise would not be diagnosed using UACR [27]. Here, we comprehensively screened the association of genetic variants on RAB38 with UPCR in DN patients and investigated the influence of gender on the association between genetic polymorphisms and UPCR. Three polymorphisms of RAB38 demonstrated significance with UPCR levels in DN patients, which are rs1027027, rs302647, and rs302646. In rs1027027, the increment of A allele increases with the expression level of UPCR. Meanwhile, the increment of the G allele resulted in a higher expression level of UPCR in rs302647 and rs302646. 

RAB38 encodes a member of the small Rab GTPase protein family that regulates intracellular vesicle trafficking between organelles and is highly expressed in tubuli [28]. Cells that with Rab38 knockdown expressed a lower BSA-gold particle endocytosis phenomenon when compared to control cells. Rab38-KO models also demonstrated more heavy proteinuria and albuminuria, whereas the observed phenotypes were successfully rescued in transgenic Rab38 models [29]. According to Teumer at al., the immunohistochemistry staining results of the diabetic Rab38 knockout mice showed a lower expression of cubilin and megalin, which decreased the amount of albumin reabsorption by the proximal tubule and increased urinary albumin excretion. [20]. Another study also demonstrated that disrupted Rab38 gene associates with the malabsorption of filtered albumin, possibly owing to aberrant intracellular vesicular trafficking for megalin and cubilin receptors [30]. This is consistent with our observation regarding the eQTL effect of rs1027027 and rs302646 on RAB38 expression in different tissues. The alleles at rs1027027 and rs302646 that correlated with decreased RAB38 expression are associated with elevated UPCR levels in DN patients. Contrarily, rs302647 that corresponded to the same situation was discovered with lower UPCR levels in DN patients. However, none of the significant eQTL effects were observed in renal tissue. It may be due to the limited availability of kidney tissues in the database. Further studies are needed to clarify the effect of these variants on renal tissues.

Synthesized in the liver, albumin functions as a protein carrier for a myriad of substances and also contributes to oncotic pressure in the plasma, which is necessary for proper fluid balance and homeostasis [31]. Most albumins are not able to pass through the glomerulus; those that filtered through are majorly reabsorbed via a proximal convoluted tubule, loop of Henle and distal convoluted tubule [32]. Megalin and cubilin, as multiligand endocytic receptors, bind to miscellaneous substances in the ultrafiltrate and mediate their reabsorption, including albumins. Despite the fact that albumins can bind with either receptor, it is generally considered that albumins first bind with cubilin. Then, megalin is required to proceed with internalization or endocytosis because cubilin lacks the intracellular cytoplasmic tail component, which enhances intracellular transport. Impaired RAB38 function may lead to increased albumin excretion via different mechanisms: altered intracellular vesicle transport may affect albumin reabsorption or the recycling of reabsorbed albumin back to the plasma membrane [33]. It may affect the delivery of proteins required, such as cubilin or megalin, for albumin endocytosis [34]. It may also directly cause glomerular damage leading to increased concentrations of urinary albumin. Therefore, reduced expression or loss of the cubilin–megalin complex may contribute to albuminuria of early diabetic kidney disease via reduced tubular reuptake of filtered albumin, which was supported by the observed association of rs1801239 with incident and persistent microalbuminuria in the Diabetes Control and Complications Trial and Epidemiology of Diabetes Interventions and Complications (DCCT/EDIC) study [35]. 

The male population was previously reported to be more susceptible to DN progression compared to females for type 2 DM [36]. Our study revealed a difference in the association between gender and UPCR levels for the variant of rs1027027, rs302647 and rs302646. The log-additive model reached a statistically significant threshold after multiple testing corrections; analysis for only the male population maintain significance, while no significance was found in the female subset analysis. Some studies that indicated a gender difference in prevalence and incidence for CKD and even ESRD, using eGFR as the phenotype, confounding factors include blood pressure, hormonal status, diets, smoking, alcohol intake, obesity, age and perhaps ethnicity. Males seem to be more likely to develop ESRD as there is a 10-year delay in females to progress to ESRD from CKD. Dialysis therapy was also more pervasive in men than in women [37]. Thus, male sex is likely to be more liable to renal function deterioration. 

In concordance with higher DN susceptibility toward males and lower for females in type 2 DM patients, males were characterized with low androgen levels (especially serum total testosterone); females were marked with high estrogen levels and high dehydroepiandrosterone (DHEA) levels. [38]. Postmenopausal women with lower estrogen levels but that had received estrogen replacement therapy exerted positive effects by reducing proteinuria [39]. As for men, the testosterone replacement treatment improved metabolic disorders to some extent but its impact on DN is still unavailable. This gives a hint towards the protective attribute of estrogen within females. In the current study, the genetic variants associated with higher proteinuria in DN patients, but were more apparent in a male population (Table 4 and Table 5). However, it is uncertain whether the genetic variants we found contribute to the phenomenon of worse DN status in the male sex. In addition, there are some limitations of this study. Firstly, we noticed the inconsistent results on the level of UPCR and DN susceptibility. The genetic influence of rs1027027, rs302647, and rs302646 was found in UPCR but not in DN susceptibility. The negative findings might be due to the small sample size that limits the statistical power. A larger cohort or replication studies in different populations are required to validate our findings. Secondly, we had to abandon rs3740925 because its primer fails to pass the quality control and there is no substitute SNP in this study. Thirdly, some important confounding factors are unmeasured and cannot be adjusted in the analysis, this may have a certain degree of influence when determining the genetic risk for DN.

## 5. Conclusions

In conclusion, we found that the A allele of rs1027027 and G allele of rs302647 and rs302646 were significantly associated with UPCR levels, which were susceptible to diabetic nephropathy in a Taiwanese population. Analysis stratified by gender presented that the male gender showed a statistically significant relation to the UPCR level. These findings highlight a novel genetic susceptibility to diabetic nephropathy. Further investigation in a larger sample size would be needed to validate the findings in this study.

## Figures and Tables

**Table 1:** Baseline characteristics of the study population.

Characteristics	Study Population
Total number	275
Male (%)	158 (57.45)
Female (%)	117 (42.55)
Age (mean ± SD years)	66.47 ± 13.73
BMI (mean ± SD kg/m^2^)	26.71 ± 4.67
Hypertension (%)	77.09
Smoking (%)	21.98
GFR (mean ± SD mL/min/1.73 m^2^)	53.17 ± 34.71
HbA1C (mean ± SD %)	6.99 ± 1.46
UPCR (mean ± SD mg/g)	1723.67 ± 2760.07

SD: standard deviation; BMI: body mass index; GFR: glomerular filtration rate; HbA1C: hemoglobin A1c; UPCR: urine protein and creatinine ratio.

**Table 2:** Basic characteristics of the RAB38 single-nucleotide polymorphisms (SNPs).

Position (GRCh37)	Variant	Ref	Alt	Location	African Frequency	American Frequency	European Frequency	Asian Frequency	Taiwan Biobank Frequency	Current Cohort Frequency	TWB-HWE *
Chr11:87846757	rs9144	C	T	3′-UTR	0.87	0.66	0.63	0.67	0.67	0.67	0.07
Chr11:87847063	rs1027027	C	A	3′-UTR	0.35	0.31	0.26	0.13	0.12	0.1	0.69
Chr11:87857804	rs11018529	T	C	intronic	0.39	0.49	0.63	0.44	0.45	0.57	0.94
Chr11:87860095	rs11018537	A	G	intronic	0.11	0.15	0.25	0.21	0.24	0.28	0.66
Chr11:87866122	rs7104391	G	A	intronic	0.96	0.88	0.95	0.83	0.72	0.7	0.36
Chr11:87884748	rs747571	T	C	intronic	0.41	0.34	0.34	0.17	0.17	0.17	0.15
Chr11:87905301	rs11018720	C	T	intronic	0.41	0.42	0.43	0.29	0.28	0.27	0.43
Chr11:87908346	rs302647	C	G	intronic	0.12	0.29	0.35	0.27	0.28	0.29	0.61
Chr11:87908448	rs302646	A	G	exon	0.76	0.79	0.87	0.57	0.56	0.57	0.74

* Hardy–Weinberg equilibrium in Taiwan Biobank cohort was tested using array data for rs1027027, rs11018529, and rs11018537, and sequencing data for other SNPs, where array data are not available.

**Table 3:** Association analysis between RAB38 single-nucleotide polymorphisms (SNPs) and the urine protein and creatinine ratio (UPCR).

				UPCR		Genotypic		Dominant		Recessive		Log-Additive	
Variant	Genotype	Number	(%)	Mean	SE	*p* Value	q Value	*p* Value	q Value	*p* Value	q Value	*p* Value	q Value
rs9144	TT	119	(45.9)	1535.8	231.2								
	CT	110	(42.5)	1839.6	285.3	0.648	0.648	0.454	0.584	0.783	0.881	0.675	0.759
	CC	30	(11.6)	1700.7	448.9								
rs1027027	CC	215	(81.1)	1487.4	165.3								
	CA	47	(17.7)	2605.4	549.1	**0.018**	0.081	**0.013**	0.058	0.061	0.243	**0.006**	**0.027**
	AA	3	(1.1)	4475.5	3295.8								
rs11018529	CC	88	(32.6)	1994.4	335.4								
	TC	131	(48.5)	1456.2	214.0	0.551	0.620	0.313	0.584	0.957	0.957	0.522	0.671
	TT	51	(18.9)	1918.1	394.6								
rs11018537	AA	144	(54.8)	1751.4	225.7								
	AG	92	(35.0)	1531.3	257.3	0.327	0.513	0.521	0.586	0.135	0.243	0.252	0.396
	GG	27	(10.3)	2211.7	778.8								
rs7104391	AA	127	(49.0)	1827.3	258.1								
	AG	110	(42.5)	1509.9	239.2	0.503	0.620	0.385	0.584	0.312	0.401	0.264	0.396
	GG	22	(8.5)	1149.8	297.7								
rs747571	TT	175	(69.4)	1469.0	182.2								
	TC	68	(27.0)	2166.0	401.0	0.342	0.513	0.606	0.606	0.236	0.354	0.978	0.978
	CC	9	(3.6)	1036.9	404.5								
rs11018720	CC	143	(53.2)	1470.7	201.1								
	CT	106	(39.4)	1914.8	287.3	0.306	0.513	0.432	0.584	0.135	0.243	0.210	0.396
	TT	20	(7.4)	2811.5	888.0								
rs302647	CC	131	(51.4)	1477.8	207.6								
	CG	98	(38.4)	1950.1	314.9	**0.040**	0.120	**0.028**	0.084	0.056	0.243	**0.011**	**0.033**
	GG	26	(10.2)	2365.4	697.4								
rs302646	GG	92	(34.5)	2440.0	376.9								
	AG	122	(45.7)	1404.8	197.9	**0.005**	**0.045**	**0.001**	**0.009**	0.086	0.243	**0.002**	**0.018**
	AA	53	(19.9)	1126.9	268.3								

All *p*-values were adjusted for age, sex, glomerular filtration rate, and hypertension history. Significant values (*p* < 0.05) were in bold. The q value is the false discovery rate (FDR) estimation for multi-testing.

**Table 4:** Association analysis between RAB38 single-nucleotide polymorphisms (SNPs) and the urine protein and creatinine ratio (UPCR) in the male cohort.

				UPCR		Genotypic		Dominant		Recessive		Log-Additive	
Variant	Genotype	Number	(%)	Mean	SE	*p* Value	q Value	*p* Value	q Value	*p* Value	q Value	*p* Value	q Value
rs9144	TT	71	(47.7)	2057.0	354.3								
	CT	62	(41.6)	2250.1	450.5	0.991	0.991	0.901	0.946	0.997	0.997	0.927	0.988
	CC	16	(10.7)	2596.9	748.4								
rs1027027	CC	116	(75.8)	1882.4	269.2								
	CA	35	(22.9)	3427.3	685.3	**0.014**	0.063	**0.015**	0.068	**0.036**	0.324	**0.006**	**0.032**
	AA	2	(1.3)	6661.1	4272.8								
rs11018529	CC	51	(32.5)	2433.1	518.7								
	TC	74	(47.1)	1955.1	340.9	0.767	0.863	0.709	0.946	0.648	0.729	0.988	0.988
	TT	32	(20.4)	2600.2	578.9								
rs11018537	AA	86	(55.8)	2294.6	343.5								
	AG	56	(36.4)	1870.2	372.1	0.248	0.558	0.537	0.946	0.095	0.339	0.236	0.531
	GG	12	(7.8)	3647.9	1588.6								
rs7104391	AA	75	(50.3)	2489.0	386.8								
	AG	65	(43.6)	1904.1	376.1	0.484	0.653	0.839	0.946	0.230	0.339	0.521	0.782
	GG	9	(6.0)	1353.3	554.6								
rs747571	TT	104	(70.3)	1915.2	281.1								
	TC	40	(27.0)	2778.6	606.9	0.508	0.653	0.946	0.946	0.264	0.339	0.767	0.986
	CC	4	(2.7)	1187.9	793.9								
rs11018720	CC	79	(51.0)	1923.2	330.9								
	CT	64	(41.3)	2426.8	418.0	0.359	0.646	0.828	0.946	0.155	0.339	0.431	0.776
	TT	12	(7.7)	4174.5	1327.0								
rs302647	CC	78	(52.3)	1841.8	319.8								
	CG	59	(39.6)	2639.6	472.0	0.052	0.156	**0.023**	0.069	0.134	0.339	**0.015**	**0.045**
	GG	12	(8.1)	3328.6	1304.0								
rs302646	GG	51	(33.3)	3497.8	595.2								
	AG	72	(47.1)	1718.7	297.3	**0.009**	0.063	**0.002**	**0.018**	0.220	0.339	**0.007**	**0.032**
	AA	30	(19.6)	1361.7	439.7								

All *p*-values were adjusted for age, glomerular filtration rate, and hypertension history. Significant values (*p* < 0.05) were in bold. The q value is the false discovery rate (FDR) estimation for multi-testing.

**Table 5:** Association analysis between *RAB38* single-nucleotide polymorphisms (SNPs) and the urine protein and creatinine ratio (UPCR) in the female cohort.

				UPCR		Genotypic		Dominant		Recessive		Log-Additive	
Variant	Genotype	Number	(%)	Mean	SE	*p* Value	q Value	*p* Value	q Value	*p* Value	q Value	*p* Value	q Value
rs9144	TT	48	(43.6)	764.9	186.8								
	CT	48	(43.6)	1309.5	286.1	0.081	0.729	0.109	0.490	0.361	0.821	0.478	0.645
	CC	14	(12.7)	676.5	270.6								
rs1027027	CC	99	(88.4)	1024.5	160.6								
	CA	12	(10.7)	208.5	60.6	0.474	0.783	0.232	0.638	0.590	0.860	0.221	0.645
	AA	1	(0.9)	104.3	0.0								
rs11018529	CC	37	(32.7)	1389.6	337.5								
	TC	57	(50.4)	808.6	185.3	0.217	0.783	0.087	0.490	0.365	0.821	0.098	0.645
	TT	19	(16.8)	769.3	267.9								
rs11018537	AA	58	(53.2)	946.0	193.0								
	AG	36	(33.0)	1004.2	297.1	0.851	0.862	0.601	0.676	0.669	0.860	0.569	0.645
	GG	15	(13.8)	1062.8	478.7								
rs7104391	AA	52	(47.3)	873.0	241.8								
	AG	45	(40.9)	940.5	191.9	0.862	0.862	0.590	0.676	0.922	0.966	0.659	0.659
	GG	13	(11.8)	1008.8	341.3								
rs747571	TT	71	(68.3)	815.5	150.9								
	TC	28	(26.9)	1290.8	401.1	0.501	0.783	0.369	0.638	0.655	0.860	0.573	0.645
	CC	5	(4.8)	916.1	450.1								
rs11018720	CC	64	(56.1)	912.1	165.4								
	CT	42	(36.8)	1134.6	316.2	0.714	0.862	0.425	0.638	0.966	0.966	0.511	0.645
	TT	8	(7.0)	766.9	452.5								
rs302647	CC	53	(50.0)	942.1	184.9								
	CG	39	(36.8)	906.9	272.6	0.395	0.783	0.729	0.729	0.176	0.821	0.370	0.645
	GG	14	(13.2)	1539.8	624.6								
rs302646	GG	41	(36.0)	1124.2	310.5								
	AG	50	(43.9)	952.7	210.8	0.522	0.783	0.398	0.638	0.303	0.821	0.263	0.645
	AA	23	(20.2)	820.6	228.4								

All *p*-values were adjusted for age, glomerular filtration rate, and hypertension history. Significant values (*p* < 0.05) were in bold. The q value is the false discovery rate (FDR) estimation for multi-testing.

## Supplementary Materials

The following are available online at https://www.mdpi.com/2075-4426/10/4/184/s1, Figure S1: Pairwise LD map of RAB38 in Han Chinese Beijing (CHB), Figure S2: Distribution of the lnUPCR in DN patients with different genotypes at rs1027027 (A), rs302647 (B) and rs302646 (C), Table S1: Association of rs1027027 with diabetic nephropathy susceptibility, Table S2: Association of rs302647 with diabetic nephropathy susceptibility, Table S3: Association of rs302646 with diabetic nephropathy susceptibility, Table S4: Expression Quantitative Trail Loci (eQTL) effects of UPCR-associated variants.

## References

[1] Williams R., Colagiuri S., Almutairi R., Montoya P.A., Basit A., Beran D., Besançon S., Bommer C., Borgnakke W., Boyko E. (2019). IDF Diabetes Atlas.

[2] Hwang S.J., Tsai J.C., Chen H.C. (2010). Epidemiology, impact and preventive care of chronic kidney disease in Taiwan. Nephrology.

[3] Fineberg D., Jandeleit-Dahm K.A., Cooper M.E. (2013). Diabetic nephropathy: Diagnosis and treatment. Nat. Rev. Endocrinol..

[4] Badal S.S., Danesh F.R. (2014). New insights into molecular mechanisms of diabetic kidney disease. Am. J. Kidney Dis..

[5] Wasser W.G., Tzur S., Wolday D., Adu D., Baumstein D., Rosset S., Skorecki K. (2012). Population genetics of chronic kidney disease: The evolving story of APOL1. J. Nephrol..

[6] Nagib A.M., Matter Y.E., Gheith O.A., Refaie A., Othman N.F., Al-Otaibi T. (2019). Diabetic Nephropathy Following Posttransplant Diabetes Mellitus. Exp. Clin. Transplant..

[7] Brennan E., McEvoy C., Sadlier D., Godson C., Martin F. (2013). The genetics of diabetic nephropathy. Genes.

[8] Wei L., Xiao Y., Li L., Xiong X., Han Y., Zhu X., Sun L. (2018). The Susceptibility Genes in Diabetic Nephropathy. Kidney Dis..

[9] Gnudi L., Coward R.J.M., Long D.A. (2016). Diabetic Nephropathy: Perspective on Novel Molecular Mechanisms. Trends Endocrinol. Metab..

[10] Majumder S., Advani A. (2017). VEGF and the diabetic kidney: More than too much of a good thing. J. Diabetes Complicat..

[11] Seaquist E.R., Goetz F.C., Rich S., Barbosa J. (1989). Familial clustering of diabetic kidney disease. Evidence for genetic susceptibility to diabetic nephropathy. N. Engl. J. Med..

[12] Imperatore G., Knowler W.C., Pettitt D.J., Kobes S., Bennett P.H., Hanson R.L. (2000). Segregation analysis of diabetic nephropathy in Pima Indians. Diabetes.

[13] Andersen A.R., Christiansen J.S., Andersen J.K., Kreiner S., Deckert T. (1983). Diabetic nephropathy in Type 1 (insulin-dependent) diabetes: An epidemiological study. Diabetologia.

[14] Krolewski A.S., Warram J.H., Christlieb A.R., Busick E.J., Kahn C.R. (1985). The changing natural history of nephropathy in type I diabetes. Am. J. Med..

[15] Van Zuydam N.R., Ahlqvist E., Sandholm N., Deshmukh H., Rayner N.W., Abdalla M., Ladenvall C., Ziemek D., Fauman E., Robertson N.R. (2018). A Genome-Wide Association Study of Diabetic Kidney Disease in Subjects With Type 2 Diabetes. Diabetes.

[16] Sandholm N., Van Zuydam N., Ahlqvist E., Juliusdottir T., Deshmukh H.A., Rayner N.W., Di Camillo B., Forsblom C., Fadista J., Ziemek D. (2017). The Genetic Landscape of Renal Complications in Type 1 Diabetes. J. Am. Soc. Nephrol..

[17] Iyengar S.K., Sedor J.R., Freedman B.I., Kao W.H., Kretzler M., Keller B.J., Abboud H.E., Adler S.G., Best L.G., Bowden D.W. (2015). Genome-Wide Association and Trans-ethnic Meta-Analysis for Advanced Diabetic Kidney Disease: Family Investigation of Nephropathy and Diabetes (FIND). PLoS Genet..

[18] Regele F., Jelencsics K., Shiffman D., Pare G., McQueen M.J., Mann J.F., Oberbauer R. (2015). Genome-wide studies to identify risk factors for kidney disease with a focus on patients with diabetes. Nephrol. Dial. Transplant..

[19] Pezzolesi M.G., Poznik G.D., Mychaleckyj J.C., Paterson A.D., Barati M.T., Klein J.B., Ng D.P., Placha G., Canani L.H., Bochenski J. (2009). Genome-wide association scan for diabetic nephropathy susceptibility genes in type 1 diabetes. Diabetes.

[20] Teumer A., Tin A., Sorice R., Gorski M., Yeo N.C., Chu A.Y., Li M., Li Y., Mijatovic V., Ko Y.A. (2016). Genome-wide Association Studies Identify Genetic Loci Associated With Albuminuria in Diabetes. Diabetes.

[21] Rule A.D., Larson T.S., Bergstralh E.J., Slezak J.M., Jacobsen S.J., Cosio F.G. (2004). Using serum creatinine to estimate glomerular filtration rate: Accuracy in good health and in chronic kidney disease. Ann. Intern. Med..

[22] Benjamini Y., Liu W. (1999). A step-down multiple hypotheses testing procedure that controls the false discovery rate under independence. J. Stat. Plan. Inference.

[23] Johnson J.L., Abecasis G.R. (2017). GAS Power Calculator: Web-based power calculator for genetic association studies. bioRxiv.

[24] Lin K.D., Hsu C.C., Ou H.Y., Wang C.Y., Chin M.C., Shin S.J. (2019). Diabetes-related kidney, eye, and foot disease in Taiwan: An analysis of nationwide data from 2005 to 2014. J. Formos. Med. Assoc..

[25] Fisher H., Hsu C.Y., Vittinghoff E., Lin F., Bansal N. (2013). Comparison of associations of urine protein-creatinine ratio versus albumin-creatinine ratio with complications of CKD: A cross-sectional analysis. Am. J. Kidney Dis..

[26] Ying T., Clayton P., Naresh C., Chadban S. (2018). Predictive value of spot versus 24-hour measures of proteinuria for death, end-stage kidney disease or chronic kidney disease progression. BMC Nephrol..

[27] Cassia M.A., Pozzi F.E., Bascapè S., Saggiante L., Daminelli G., Cirelli C., Andi P.T.D., Elli M., Gallieni M. (2016). Proteinuria and Albuminuria at Point of Care. Nephrol. Point Care.

[28] Stenmark H. (2009). Rab GTPases as coordinators of vesicle traffic. Nat. Rev. Mol. Cell Biol..

[29] Rangel-Filho A., Lazar J., Moreno C., Geurts A., Jacob H.J. (2013). Rab38 modulates proteinuria in model of hypertension-associated renal disease. J. Am. Soc. Nephrol..

[30] Tojo A., Onozato M.L., Ha H., Kurihara H., Sakai T., Goto A., Fujita T., Endou H. (2001). Reduced albumin reabsorption in the proximal tubule of early-stage diabetic rats. Histochem. Cell Biol..

[31] Levitt D.G., Levitt M.D. (2016). Human serum albumin homeostasis: A new look at the roles of synthesis, catabolism, renal and gastrointestinal excretion, and the clinical value of serum albumin measurements. Int. J. Gen. Med..

[32] Tojo A., Kinugasa S. (2012). Mechanisms of glomerular albumin filtration and tubular reabsorption. Int. J. Nephrol..

[33] Bultema J.J., Di Pietro S.M. (2013). Cell type-specific Rab32 and Rab38 cooperate with the ubiquitous lysosome biogenesis machinery to synthesize specialized lysosome-related organelles. Small GTPases.

[34] Christensen E.I., Devuyst O., Dom G., Nielsen R., Van der Smissen P., Verroust P., Leruth M., Guggino W.B., Courtoy P.J. (2003). Loss of chloride channel ClC-5 impairs endocytosis by defective trafficking of megalin and cubilin in kidney proximal tubules. Proc. Natl. Acad. Sci. USA.

[35] Böger C.A., Chen M.H., Tin A., Olden M., Köttgen A., de Boer I.H., Fuchsberger C., O’Seaghdha C.M., Pattaro C., Teumer A. (2011). CUBN is a gene locus for albuminuria. J. Am. Soc. Nephrol..

[36] Radcliffe N.J., Seah J.M., Clarke M., MacIsaac R.J., Jerums G., Ekinci E.I. (2017). Clinical predictive factors in diabetic kidney disease progression. J. Diabetes Investig..

[37] Iseki K. (2008). Gender differences in chronic kidney disease. Kidney Int..

[38] Wang C., Zhang W., Wang Y., Wan H., Chen Y., Xia F., Zhang K., Wang N., Lu Y. (2019). Novel associations between sex hormones and diabetic vascular complications in men and postmenopausal women: A cross-sectional study. Cardiovasc. Diabetol..

[39] Clotet S., Riera M., Pascual J., Soler M.J. (2016). RAS and sex differences in diabetic nephropathy. Am. J. Physiol. Renal Physiol..

