# Supplementary material for: Gender Differences in Genetic Associations of RAB38 with Urinary Protein-to-Creatinine Ratio (UPCR) Levels in Diabetic Nephropathy Patients
Source: J Pers Med. 2020 Oct 21;10(4):184. doi: 10.3390/jpm10040184 (PMC7711808; doi:10.3390/jpm10040184)
Supplement: Supplementary file 1 [file jpm-10-00184-s001.pdf]

Supplementary Material

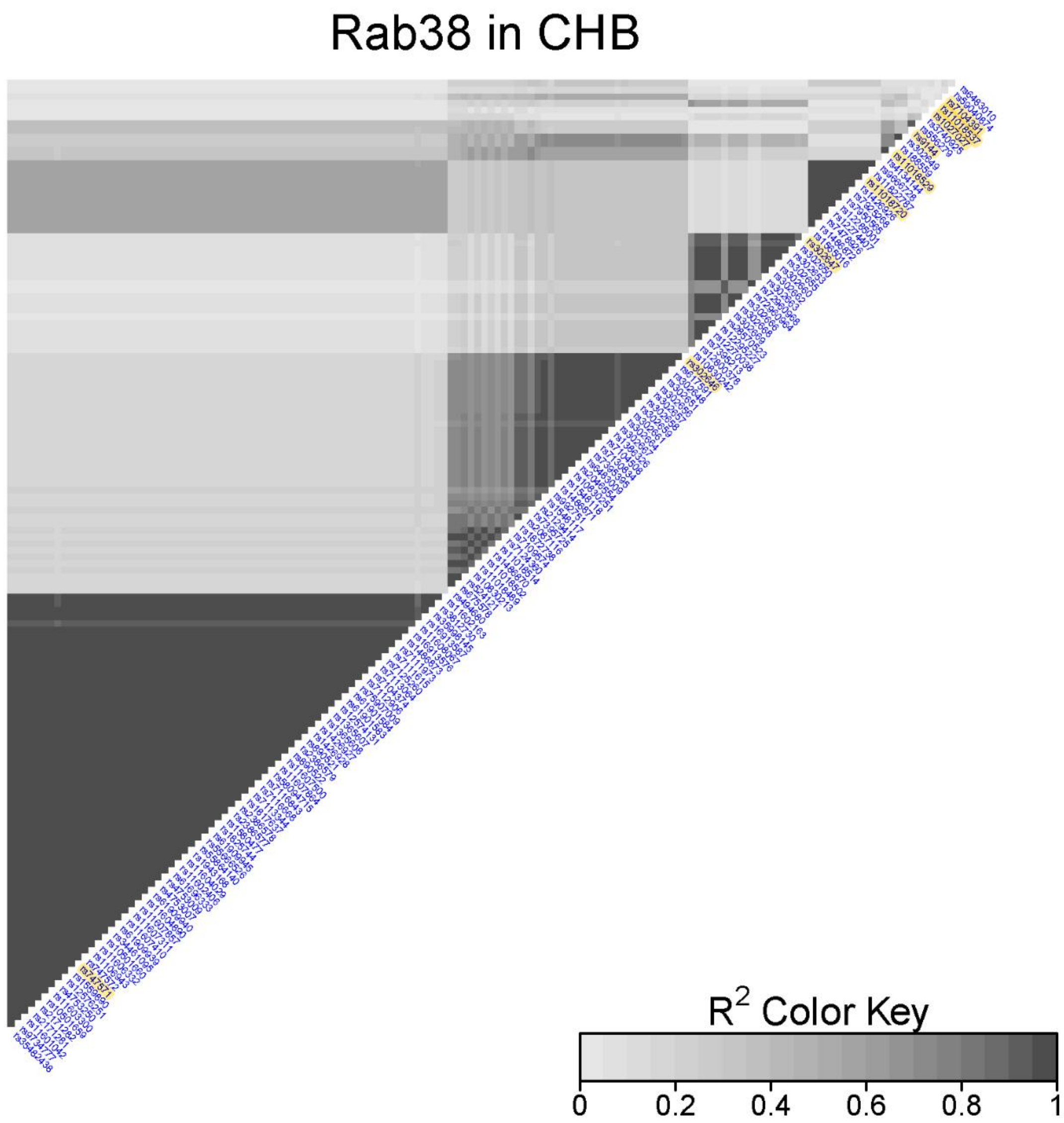

Figure S1: Pairwise LD map of *RAB38* in Han Chinese Beijing (CHB).
SNPs genotyped in this study were highlighted in yellow.

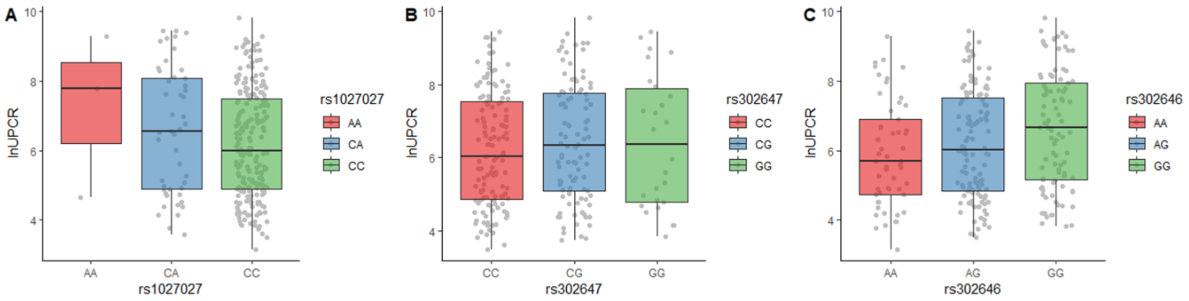

Figure S2: Distribution of the lnUPCR in DN patients with different genotypes at rs1027027 (A), rs302647 (B) and rs302646 (C)

Table S1: Association of rs1027027 with diabetic nephropathy susceptibility.

Model	Genotype	Number (%) (n=265)	TWB[a] (%) (n=24625)	OR (95% CI)	*P* value
Genotypic	AA	3 (1.1)	357 (1.4)	0.75 (0.15-2.23)	0.802
	AC	47 (17.7)	5162 (21.0)	0.81 (0.58-1.12)	0.198
	CC	215 (81.1)	19106 (77.6)	Reference	—
Dominant	AA+AC	50 (18.9)	5519 (22.4)	0.81 (0.58-1.10)	0.182
	CC	215 (81.1)	19106 (77.6)	Reference	—
Recessive	AA	3 (1.1)	357 (1.4)	0.78 (0.16-2.32)	1.000
	AC+CC	262 (98.9)	24268 (98.6)	Reference	—
Allelic	A	53 (10.0)	5876 (11.93)	0.820 (0.60-1.09)	0.200
	C	477 (90.0)	43374 (88.07)	Reference	

[a]General population from Taiwan Biobank. All apply Fisher's exact test. Calculates odds ratio by conditional maximum likelihood estimation (Fisher).

Table S2: Association of rs302647 with diabetic nephropathy susceptibility.

Model	Genotype	Number (%) (n=255)	TWB[a] (%) (n=1000)	OR (95% CI)	*P* value
Genotypic	GG	26 (10.2)	76 (7.6)	1.34 (0.79-2.21)	0.241
	CG	98 (38.4)	411 (41.1)	0.93 (0.69-1.26)	0.656
	CC	131 (51.4)	513 (51.3)	Reference	—
Dominant	GG+CG	124 (48.6)	487 (48.7)	1.00 (0.75-1.33)	1.000
	CC	131 (51.4)	513 (51.3)	Reference	—
Recessive	GG	26 (10.2)	76 (7.6)	1.38 (0.83-2.24)	0.198
	GC+CC	229 (89.8)	924 (92.4)	Reference	—
Allelic	G	150 (29.4)	563 (28.2)	1.06 (0.85-1.32)	0.583
	C	360 (70.6)	1437 (71.9)	Reference	

[a]General population from Taiwan Biobank. All apply Fisher's exact test.

Calculates odds ratio by conditional maximum likelihood estimation (Fisher). Confidence intervals are calculated using exact methods (Fisher).

Table S3: Association of rs302646 with diabetic nephropathy susceptibility.

Model	Genotype	Number (%) (n=267)	TWB[a] (%) (n=1000)	OR (95% CI)	*P* value
Genotypic	GG	92 (34.5)	315 (31.5)	1.09 (0.73-1.63)	0.699
	AG	122 (45.6)	488 (48.8)	0.93 (0.64-1.36)	0.710
	AA	53 (19.9)	197 (19.7)	Reference	—
Dominant	GG+AG	214 (80.1)	803 (80.3)	0.99 (0.70-1.42)	1.000
	AA	53 (19.9)	197 (19.7)	Reference	—
Recessive	GG	92 (34.5)	315 (31.5)	1.14 (0.85-1.53)	0.376
	AG+AA	175 (65.5)	685 (68.5)	Reference	—
Allelic	G	306 (57.3)	1118 (55.9)	1.06 (0.87-1.29)	0.589
	A	228 (42.7)	882 (44.1)	Reference	

[a]General population from Taiwan Biobank. All apply Fisher's exact test.

Calculates odds ratio by conditional maximum likelihood estimation (Fisher). Confidence intervals are calculated using exact methods (Fisher).

Table S4: Expression Quantitative Trail Loci (eQTL) effects of UPCR-associated variants

SNP Id	Ref	Alt	Gene Symbol	P-Value	NES[a]	Tissue
rs1027027	C	A	RAB38	1.00E-04	-0.12	Esophagus - Mucosa
rs302647	C	G	RAB38	5.50E-25	0.43	Esophagus - Muscularis
rs302647	C	G	RAB38	1.30E-18	0.49	Esophagus - Gastroesophageal Junction
rs302647	C	G	RAB38	3.10E-16	0.37	Adipose - Subcutaneous
rs302647	C	G	RAB38	1.10E-15	0.47	Colon - Transverse
rs302647	C	G	RAB38	3.30E-15	0.25	Nerve - Tibial
rs302647	C	G	RAB38	7.10E-14	0.3	Cells - Cultured fibroblasts
rs302647	C	G	RAB38	6.30E-13	0.36	Heart - Atrial Appendage
rs302647	C	G	RAB38	9.70E-13	0.3	Lung
rs302647	C	G	RAB38	7.30E-12	0.34	Muscle - Skeletal
rs302647	C	G	RAB38	9.00E-12	0.31	Whole Blood
rs302647	C	G	RAB38	5.40E-11	0.12	Skin - Sun Exposed (Lower leg)
rs302647	C	G	RAB38	8.70E-11	0.34	Colon - Sigmoid
rs302647	C	G	RAB38	1.00E-09	0.63	Liver
rs302647	C	G	RAB38	2.20E-09	0.22	Artery - Tibial
rs302647	C	G	RAB38	4.70E-09	0.26	Adipose - Visceral (Omentum)
rs302647	C	G	RAB38	6.90E-09	0.27	Artery - Aorta
rs302647	C	G	RAB38	1.30E-08	0.35	Heart - Left Ventricle
rs302647	C	G	RAB38	2.20E-07	0.24	Breast - Mammary Tissue
rs302647	C	G	RAB38	7.20E-07	0.29	Stomach
rs302647	C	G	RAB38	9.90E-07	0.11	Skin - Not Sun Exposed (Suprapubic)
rs302647	C	G	RAB38	6.90E-06	0.38	Brain - Nucleus accumbens (basal ganglia)
rs302647	C	G	RAB38	9.40E-06	0.33	Spleen
rs302647	C	G	RAB38	1.00E-05	0.35	Ovary
rs302646	A	G	RAB38	8.80E-13	-0.2	Skin - Not Sun Exposed (Suprapubic)
rs302646	A	G	RAB38	6.00E-11	-0.16	Skin - Sun Exposed (Lower leg)
rs302646	A	G	RAB38	4.80E-09	-0.24	Esophagus - Mucosa

[a]NES, normalized effect size.
